# Roles of ATP Hydrolysis by FtsEX and Interaction with FtsA in Regulation of Septal Peptidoglycan Synthesis and Hydrolysis

**DOI:** 10.1128/mBio.01247-20

**Published:** 2020-07-07

**Authors:** Shishen Du, Sebastien Pichoff, Joe Lutkenhaus

**Affiliations:** a Department of Microbiology, College of Life Sciences, Wuhan University, Wuhan, China; b Department of Microbiology, Molecular Genetics and Immunology, University of Kansas Medical Center, Kansas City, Kansas, USA; Max Planck Institute for Terrestrial Microbiology

**Keywords:** amidase, EnvC, FtsA, FtsEX, peptidoglycan

## Abstract

In Escherichia coli, FtsEX coordinates peptidoglycan (PG) synthesis and hydrolysis at the septum. It acts on FtsA in the cytoplasm to promote recruitment of septal PG synthetases and recruits EnvC, an activator of septal PG hydrolases, in the periplasm. Following recruitment, ATP hydrolysis by FtsEX is thought to regulate both PG synthesis and hydrolysis, but how it does this is not well understood. Here, we show that an ATPase mutant of FtsEX blocks septal PG synthesis similarly to cephalexin, suggesting that ATP hydrolysis by FtsEX is required throughout septation. Using mutants that uncouple the roles of FtsEX in septal PG synthesis and hydrolysis, we find that recruitment of EnvC to the septum by FtsEX, but not ATP hydrolysis, is required to promote cell separation when the NlpD-mediated cell separation system is present. However, ATP hydrolysis by FtsEX becomes necessary for efficient cell separation when the NlpD system is inactivated, suggesting that the ATPase activity of FtsEX is required for optimal activity of EnvC. Importantly, under conditions that suppress the role of FtsEX in cell division, disruption of the FtsEX-FtsA interaction delays cell separation, highlighting the importance of this interaction in coupling the cell separation system with the septal PG synthetic complex.

## Abstract (precis)

Cytokinesis in Gram-negative bacteria requires coordinated invagination of the three layers of the cell envelope; otherwise, cells become sensitive to hydrophobic antibiotics and can even undergo cell lysis. *In* E. coli, the ABC transporter FtsEX couples the synthesis and hydrolysis of the stress-bearing peptidoglycan layer at the septum by interacting with FtsA and EnvC, respectively. ATP hydrolysis by FtsEX is critical for its function, but the reason why is not clear. Here, we find that in the absence of ATP hydrolysis, FtsEX blocks septal PG synthesis similarly to cephalexin. However, an FtsEX ATPase mutant, under conditions where it cannot block division, rescues *ftsEX* phenotypes as long as a partially redundant cell separation system is present. Furthermore, we find that the FtsEX-FtsA interaction is important for efficient cell separation.

## INTRODUCTION

For most bacteria, cell division requires the remodeling of peptidoglycan (PG) at the division site, which entails the coordinated activation of PG synthetases and hydrolases (1). These enzymes need to be highly regulated, since dysregulation could lead to a breach in the wall and to cell lysis due to turgor pressure. Spatial and temporal regulation of these enzymes is achieved by coupling their recruitment and activation to the Z ring (2). Septal peptidoglycan synthesis is carried out by FtsW, a member of the SEDS family, which was recently shown to have PG glycosyltransferase activity (3, 4), and FtsI, a transpeptidase (5, 6). The FtsWI complex is recruited to the Z ring by the FtsQLB complex and in Escherichia coli is activated by the arrival of FtsN (7). A theme that has emerged from work with E. coli and Streptococcus pneumoniae is that peptidoglycan hydrolases or their activators are recruited to the division site by FtsEX, a member of the ABC transporter family. In E. coli, FtsEX recruits EnvC, which activates amidases (AmiA and AmiB) at the Z ring (8), whereas in S. pneumoniae, FtsEX directly recruits and regulates the PcsB hydrolase (9). Following these pioneering studies, FtsEX was also found to regulate the RipC hydrolase in Mycobacterium tuberculosis and Corynebacterium glutamicum (10, 11).

FtsEX is a member of the type VII subfamily of ABC transporters, which employ a mechanotransduction mechanism to perform work in the periplasm (12). Members of this subfamily include MacB, which expels antibiotics and virulence factors, and LolCDE, which extracts lipoproteins from the outer leaflet of the cytoplasmic membrane in Gram-negative bacteria (13). In E. coli, FtsEX is essential for cell division at low to moderate osmolarity and plays a role in cell separation (8, 14, 15). The complex localizes to the Z ring as it forms through an interaction between FtsE and the conserved tail (conserved C-terminal peptide [CCTP]) of FtsZ (16). In a step that does not require ATP hydrolysis, FtsEX (i) interacts with FtsA to promote the recruitment of downstream division proteins and (ii) recruits EnvC through an interaction between the large periplasmic loop of FtsX and the coiled-coil domain of EnvC (8, 17). Once the divisome is assembled, FtsEX must undergo ATP hydrolysis; otherwise, it blocks septal PG synthesis through its interaction with FtsA (17). In addition to relieving this block, ATP hydrolysis by FtsEX is thought to cause a conformational change in EnvC, which allows it to activate AmiB and AmiA at the division site to remove cross-links between the glycan strands, leading to cell separation (18).

Mutations in *ftsE* that affect ATP binding or hydrolysis have been shown to impair cell division and cell separation (8, 15, 17). However, it is not clear how ATP hydrolysis is regulated. In the case of MacB, its binding partner in the periplasm, MacA, greatly stimulates its ATPase activity (19). By analogy, EnvC would stimulate the ATPase activity of FtsEX, allowing cell division to proceed. However, an FtsEX^Δlp^ mutant, carrying a deletion in the large periplasmic loop that eliminates interaction with EnvC, supports cell division (17), indicating that it is able to carry out ATP hydrolysis.

Despite its important roles in cell division, *ftsEX* can be deleted in E. coli under a variety of conditions, including increased osmolarity, overexpression of *ftsQAZ*, and mutations in *ftsA* that reduce FtsA’s self-interaction (17, 20, 21). These suppressive conditions appear to enhance the interaction between FtsA and FtsN so that the divisome is assembled and activated (22). However, EnvC is not recruited to the Z ring, and as a consequence, *ΔftsEX* cells display a mild chaining phenotype (8). The length of these chains is limited, since cells separate due to a partially redundant pathway involving another amidase (AmiC) controlled by NlpD (18). The activities of these two systems overlap, and severe chaining is observed only when both systems are inactive, as when the activators (*ftsEX* or *envC* and *nlpD*) or all three amidases are deleted (8, 23).

In this study, we further explore how FtsEX coordinates septal PG synthesis and hydrolysis. We find that an ATPase mutant of FtsEX mimics cephalexin in blocking division, suggesting that ATP hydrolysis by FtsEX is required throughout the septation process. We also find that the loss of *ftsEX*, like the loss of EnvC (24), results in sensitivity to hydrophobic antibiotics. However, this sensitivity is suppressed by an ATPase mutant of FtsEX, which also suppresses chaining, suggesting that EnvC can promote cell separation in the absence of ATPase hydrolysis. However, in the absence of the other cell separation system, the ATPase mutant of FtsEX only partially rescues cell separation, indicating that ATP hydrolysis by FtsEX is required for optimal activity of EnvC. In addition, we find that FtsEX must interact with FtsA for cells to separate efficiently, suggesting that tight coupling of the cell separation system with the septal PG synthetic machinery is critical for timely cell separation.

## RESULTS

### An ATPase mutant of FtsE blocks ongoing septal PG synthesis.

**FIG 1:**
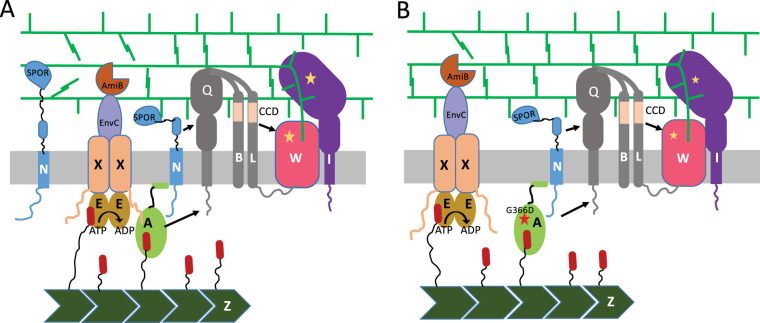
Model for the role of FtsEX in coupling septal PG synthesis with hydrolysis. (A) FtsEX interacts with FtsA to couple septal PG synthesis and hydrolysis. FtsEX is recruited to the Z ring by the interaction of FtsE with the tail of FtsZ. Once at the ring, FtsEX interacts with FtsA to promote the recruitment of other division proteins and directly recruits EnvC to regulate septal PG synthesis and hydrolysis. The FtsEX-FtsA interaction, which involves the N-terminal domain of FtsX and residue G366 in FtsA, links the cell separation system (FtsEX-EnvC-AmiB/AmiA) to the septal PG synthesis machinery (FtsA-FtsQLB-FtsWI), leading to efficient cell division and cell separation. FtsN activates FtsWI by signaling through FtsQLB (involving the CCD domains of FtsLB) and FtsA. EnvC at the Z ring activates amidases (AmiA and AmiB) to remove cross-links between glycan strands for cell separation. In the absence of ATP hydrolysis, FtsEX is able to promote EnvC-dependent cell separation activity, which is sufficient as long as the NlpD-regulated system is present. However, the hydrolysis-independent activity is not sufficient when NlpD is deleted. Additional FtsN is recruited through the binding of denuded PG by the SPOR domain, which further stimulates PG synthesis. (B) Loss of the FtsA-FtsX interaction uncouples septal PG synthesis and hydrolysis. In the absence of the FtsEX-FtsA interaction (due to the presence of FtsA*^,G366D^), FtsEX is still associated with the Z ring (FtsE-FtsZ tail interaction); however, it is no longer tethered to FtsA, decreasing the efficiency of this cell separation pathway. This lack of coupling results in less-efficient removal of the stem peptides connecting the glycan strands, delaying FtsN recruitment and cell separation. This deficiency can be overcome by increasing the level of FtsEX and is more evident in the absence of NlpD (Fig. 7). Most Fts proteins are indicated by a single letter. The diagram is simplified by omitting FtsK (which probably links FtsQ to FtsA) and ZipA, an additional membrane tether for FtsZ filaments.

In the current model for divisome activation, FtsN triggers septal PG synthesis by acting on FtsA and FtsQLB (25–27). Once septal PG synthesis starts, EnvC in the periplasm stimulates AmiA and AmiB to sever peptide cross-links in the newly synthesized PG, generating denuded peptidoglycan chains. Additional FtsN is attracted to the septum through the binding of these chains by the SPOR domain of FtsN, further enhancing septal PG synthesis (7) (Fig. 1A). Thus, FtsN activation can be divided into the triggering step and the self-enhancement stage. Previous results (17) showed that the ATPase mutant FtsE^D162N^X (which, by analogy with MacB, would bind ATP but be defective in ATP hydrolysis [12]) acts on FtsA to generate smooth filaments, indicating that it blocks the initiation of constriction. However, it is not clear if FtsE^D162N^X just blocks initiation or if it blocks ongoing septation as well (17).

**FIG 2:**
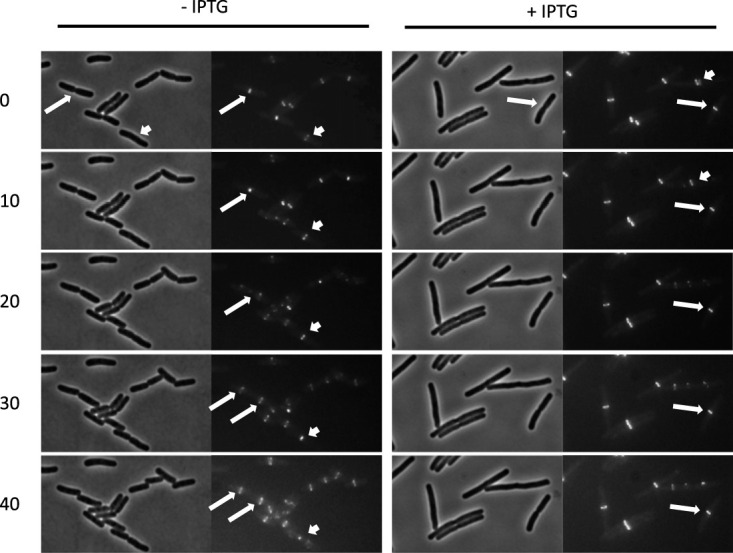
Expression of FtsE^D162N^X blocks ongoing constrictions. An overnight culture of HC261 (*zapA-GFP*)/pSD221-D162N (pEXT22 *P*_tac_::*ftsE^D162N^X*) was diluted 100-fold in LB with sucrose and antibiotics and was grown at 30°C. After 2 h, 2 μl of the culture was spotted onto a 2% agarose pad containing LB. ZapA-GFP and cell division were followed for 40 min. To follow ZapA-GFP localization in cells expressing FtsE^D162N^X, the overnight culture of HC261 (*zapA*::*GFP*)/pSD221-D162N was diluted 100-fold in LB with sucrose and antibiotics and was grown at 30°C until the OD_600_ reached about 0.6. The culture was diluted 5 times in the same medium containing 250 μM IPTG. After induction for 1 h, 2 μl of the culture was spotted onto a 2% agarose pad containing LB with IPTG and sucrose and was monitored for 40 min. Long arrows in the left panel indicate a cell with a constriction at time zero which is completed by 20 min, with new Z rings forming in the daughter cells by 30 min. The other arrow indicates a ring that shrinks in diameter during the period of observation. The long arrow in the right panel indicates a cell with a constriction and an associated Z ring that does not change diameter during the 40 min of the experiment. The other arrow indicates a rare example of a cell with a Z ring that disappears around 20 min.

To determine the effect of FtsE^D162N^X on ongoing septation, we monitored the contraction of Z rings and division upon expression of *ftsE^D162N^X.* We introduced a plasmid expressing *ftsE^D162N^X* under the control of isopropyl-β-d-thiogalactopyranoside (IPTG) into a strain that constitutively expresses *zapA-gfp* from the chromosome as a proxy for Z rings. One hour after the addition of ITPG to an exponential-phase culture, a sample was spotted onto an agarose pad (with IPTG) and followed by time-lapse microscopy at room temperature for 40 min (Fig. 2; see also Fig. S1 in the supplemental material). In the control sample without IPTG, all cells with a visible constriction at time zero had completed constriction by 40 min (18/18), and this was accompanied by the disappearance of the associated Z rings and the appearance of new Z rings in the daughter cells (Fig. S1; Table S1). An example of such a cell is indicated by long arrows in Fig. 2. In other cells, a Z ring was present without an apparent constriction at time zero (short arrows), but by 40 min, the constriction was almost complete: the Z ring was reduced to a spot, and the daughter cells were nearly separated.

10.1128/mBio.01247-20.1 FIG S1 Expression of FtsE^D162N^X blocks both the initiation of constriction and ongoing constrictions. The cells in the white dotted rectangles are shown and analyzed in Fig. 2. Only the cells at the beginning and end of the time-lapse analysis from Fig. 2 are shown. Cells with a visible constriction are numbered for the sample with IPTG: red triangles indicate cells with completed constrictions. Scale bar, 5 μm. Download FIG S1, PDF file, 0.3 MB. Copyright © 2020 Du et al. 2020 Du et al. This content is distributed under the terms of the Creative Commons Attribution 4.0 International license.

10.1128/mBio.01247-20.3 TABLE S1 Z ring formation and cell constriction in cells with or without FtsE^D162N^X overproduction. Download Table S1, DOCX file, 0.01 MB. Copyright © 2020 Du et al. 2020 Du et al. This content is distributed under the terms of the Creative Commons Attribution 4.0 International license.

In the sample in which FtsE^D162N^X was induced (with IPTG added), the cells had increased in length by 1 h after IPTG addition (time zero), a finding consistent with FtsE^D162N^X blocking division. In cells without a constriction, the Z ring persisted but did not contract during the 40 min of observation (Fig. 2 and Fig. S1). Even in cells with a visible constriction (long arrow) at time zero, the Z ring persisted without noticeably changing in diameter (7/11). In four constricting cells, the Z ring disappeared, but in these cells the constriction was already very deep at time zero, suggesting that very late stages of cell division may not be blocked (Fig. S1 and Table S1). In rare cases (2/46), the Z ring disappeared without a constriction (Fig. 2, short arrow). These results indicate that FtsE^D162N^X blocks ongoing constriction without disrupting the Z ring.

**FIG 3:**
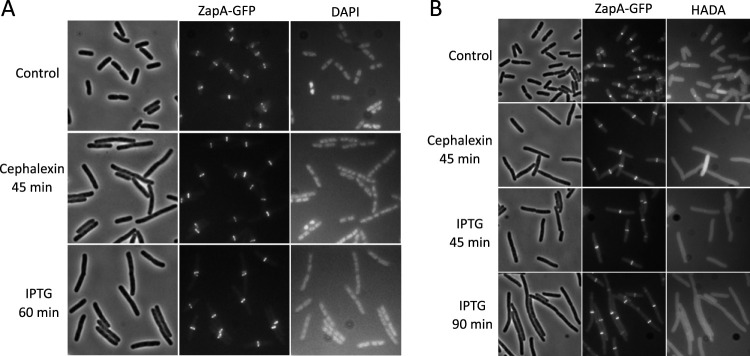
Overexpression of FtsE^D162N^X phenocopies cephalexin in blocking septal PG synthesis. (A) Overexpression of FtsE^D162N^X does not disrupt Z rings or affect nucleoid segregation. An overnight culture of HC261/pSD221-D162N (pEXT22 *P*_tac_::*ftsE^D162N^X*) was grown to exponential phase in LB sucrose medium at 30°C. The culture was then diluted 5-fold in the same medium with or without the addition of cephalexin (20 μg/ml) or IPTG (1 mM). The cultures were then grown at 30°C for another 45 min or 60 min, respectively, stained with DAPI for 5 min, and fixed with paraformaldehyde and glutaraldehyde. (B) Overexpression of FtsE^D162N^X blocks incorporation of HADA. ZapA-GFP localization and HADA labeling were examined in cells treated with cephalexin or following the expression of FtsE^D162N^X. Cells were grown as in the experiment for which results are shown in panel A for 45 min, and a 200-μl sample was then taken from each culture and incubated with 2 μl of HADA in DMSO (final concentration, 0.25 mM) for 1 min. The cells were immediately fixed with paraformaldehyde and glutaraldehyde for 15 min on ice and were then washed four times with PBS. The cells were then resuspended in 50 μl of PBS and spotted onto an agarose pad for imaging. A sample from the culture with IPTG was also taken at 90 min.

The block to division by FtsE^D162N^X is phenotypically similar to the block by cephalexin, which inactivates PBP3 (FtsI) and blocks both initiation and ongoing constriction events (28). To see to what extent the block by FtsE^D162N^X mimics the action of cephalexin, the two treatments were compared directly. An exponentially growing culture of the strain used in the experiments described above was split in two, and cephalexin was added to one half for 45 min, while IPTG was added to the other half for 60 min to induce FtsE^D162N^X (Fig. 3A). At these time points, the cell lengths of the two cultures were comparable, indicating that cell division was inhibited to similar extents, whereas segregation of nucleoids was not affected (Fig. 3A). Both treatments resulted in almost every cell containing a single ZapA-green fluorescent protein (GFP) ring at midcell, a finding consistent with an inability of Z rings to constrict. To confirm that septal PG synthesis was inhibited, we used the fluorescent d-amino acid HADA to label newly synthesized PG (6, 29). In the control culture, a band of HADA was observed to coincide with the position of the Z ring in all cells with a constriction (Fig. 3B). In contrast, in the cephalexin-treated culture, no band of HADA was observed to overlap the Z ring (0/154) (Table S2). Similarly, in the culture with FtsE^D162N^X induced for 45 min, bands of HADA overlapping the Z ring were infrequent (14/152) (Table S2), and they were even less frequent by 90 min (10/180) (Table S2). These results provide additional support for the observations that FtsE^D162N^X expression phenocopies cephalexin treatment and that FtsE^D162N^X blocks ongoing septation.

10.1128/mBio.01247-20.4 TABLE S2 Colocalization of Z rings and HADA in cells treated with cephalexin or overexpressing FtsE^D162N^X. Download Table S2, DOCX file, 0.01 MB. Copyright © 2020 Du et al. 2020 Du et al. This content is distributed under the terms of the Creative Commons Attribution 4.0 International license.

### An ATPase mutant of FtsEX suppresses the sensitivity of a Δ*ftsEX* mutant to hydrophobic antibiotics.

Previous studies indicated that ATP hydrolysis by FtsEX was essential for septal PG synthesis and for the activation of amidases (AmiA and AmiB) by EnvC to cleave septal PG for cell separation (8, 17). However, *in vitro* studies found that EnvC activated AmiA and AmiB in the absence of FtsEX (18), raising questions about the role of ATP hydrolysis by FtsEX in EnvC-induced activation of the amidases. Thus, we decided to reassess the role of ATP hydrolysis by FtsEX in EnvC-mediated cell separation. Because ATPase mutants of FtsEX cannot support septal PG synthesis, as shown above, we took advantage of an *ftsA* allele (ftsA*^,^*^G366D^*) that carries two mutations (17). One mutation (ftsA*) suppresses the growth defects of Δ*ftsEX* cells, but the cells are sensitive to inhibition by an FtsEX ATPase mutant (17). However, the addition of a second mutation (*ftsA^G366D^*) confers resistance to this inhibition by disrupting the FtsEX-FtsA interaction (17). Thus, the ftsA*^,^*^G366D^* allele allows us to test if ATP hydrolysis by FtsEX is required for cell separation without worrying about inhibition of septal PG synthesis. We also took advantage of the sensitivity of chaining mutants to hydrophobic drugs to devise a complementation test.

**FIG 4:**
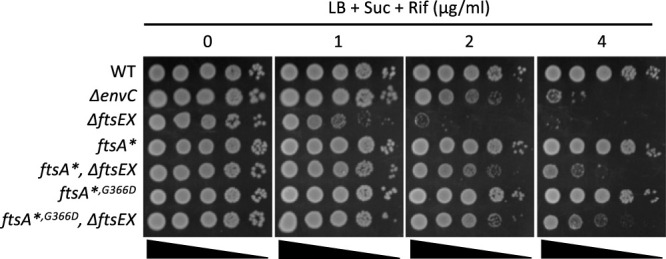
The increased sensitivity of *ΔftsEX* cells (relative to *ΔenvC* cells) to a hydrophobic drug (rifampin) is suppressed by ftsA* alleles. Cultures of the various strains were serially diluted 10-fold, and 3 μl of each dilution was spotted onto plates containing increasing concentrations of rifampin. The strains were derivatives of W3110 and included W3110 (*leu*::Tn*10*) (WT), SD208 (W3110 *envC*::*kan*), SD220 (W3110 *leu::*Tn*10 ftsEX*::*cat*), PS2343 (W3110 *leu*::Tn10 ftsA*), SD221 (W3110 *leu*::Tn10 ftsA* ftsEX::*cat*), SD249 (W3110 *leu*::Tn10 ftsA*^,^*^G366D^*), and SD262 (W3110 *leu*::Tn10 ftsA*^,^*^G366D^ ftsEX*::*cat*).

Deletion of *envC* results in a mild chaining phenotype and sensitivity to hydrophobic drugs (24, 30, 31). Since deletion of *ftsEX* also results in a mild chaining phenotype under conditions permissive for growth, we tested the sensitivity of a Δ*ftsEX* strain to rifampin, a hydrophobic drug. We found that the loss of *ftsEX*, like the loss of *envC*, displayed increased sensitivity to rifampin (Fig. 4). However, the Δ*ftsEX* strain was even more sensitive than the Δ*envC* strain; the Δ*envC* mutant was unable to form colonies on plates with 4 μg/ml of rifampin, whereas the Δ*ftsEX* mutant was unable to form colonies on plates with 2 μg/ml. The increased sensitivity of the Δ*ftsEX* mutant is accompanied by a 50% increase in the average cell length (Table S3) and is consistent with FtsEX having roles beyond regulating EnvC, as previously reported (14, 17). Introduction of the ftsA* or ftsA*^,^*^G366D^* allele into the Δ*ftsEX* mutant partially suppressed the sensitivity to rifampin such that these mutants were comparable to the Δ*envC* strain (Fig. 4). Both of these alleles also reduced the average cell length to slightly less than that of the Δ*envC* strain, indicating that they behaved similarly (Table S3).

10.1128/mBio.01247-20.5 TABLE S3 Average cell lengths of *ftsEX* and *envC* deletion strains. Download Table S3, DOCX file, 0.01 MB. Copyright © 2020 Du et al. 2020 Du et al. This content is distributed under the terms of the Creative Commons Attribution 4.0 International license.

**FIG 5:**
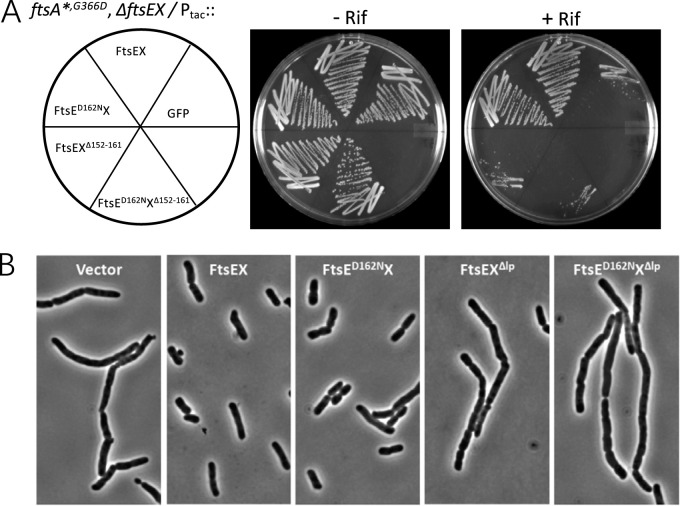
EnvC, but not ATP hydrolysis by FtsEX, is required to suppress the phenotypic defects of *ΔftsEX* cells. (A) Interaction with EnvC, but not ATPase hydrolysis, is required for FtsEX to suppress the sensitivity of a ΔftsEX ftsA*^,^*^G366D^* strain to rifampin. Sensitivity to rifampin was assessed by streaking strains onto LB plates containing 0.2 M sucrose and 4 μg/ml of rifampin. Shown are results for SD262 (*leu*::Tn10 ftsA*^,^*^G366D^ ftsEX*::*cat*) carrying derivatives of pDSW208 expressing various alleles of *ftsEX* under the control of an IPTG-inducible promoter. The plasmids were pDSW208 (P_204_::*gfp*), pSEB428 (P_204_::*ftsEX*), pSEB428-D162N (P_204_::*ftsE^D162N^X*), pSD213 (P_204_::*ftsEX^Δlp^*), and pSD213-D162N (P_204_::*ftsE^D162N^X^Δlp^*). (B) Interaction with EnvC, but not ATPase hydrolysis, is required for FtsEX to suppress the cell separation defect of the ΔftsEX ftsA*^,^*^G366D^* strain. The strains in panel A were grown to exponential phase in LB with sucrose and antibiotics, and samples were taken for photography. Note that rifampin sensitivity correlates with the chaining phenotype.

The sensitivity of the ΔftsEX ftsA*^,^*^G366D^* strain to rifampin allowed us to test whether ATP hydrolysis by FtsEX was critical for the suppression of sensitivity to this drug by EnvC. For this purpose, plasmids carrying various alleles of *ftsEX* under the control of an IPTG-inducible promoter were introduced into this strain. As shown in Fig. 5A, *ftsE^D162N^X* suppressed rifampin sensitivity as well as wild-type (WT) *ftsEX*. In contrast, neither *ftsEX^Δlp^* nor *ftsE^D162N^X^Δlp^*, which are unable to interact with EnvC due to a deletion in the large periplasmic loop of FtsX, was able to suppress the rifampin sensitivity. Thus, interaction of FtsEX with EnvC, but not its ATPase activity, is required for the suppression of sensitivity to hydrophobic drugs.

To see if rifampin sensitivity correlated with the chaining phenotype of the strains, they were grown in liquid medium to exponential phase and were examined by phase-contrast microscopy (Fig. 5B). Indeed, the ΔftsEX ftsA*^,G366D^ strain containing the vector produced elongated cells (average length, 21.1 ± 9.3 μM [Table S4]) due to cell chaining, whereas chaining was suppressed by induction of WT *ftsEX* (average length, 5.7 ± 3.8 μM [Table S4]). In agreement with the drug sensitivity test, the strain expressing *ftsE^D162N^X* displayed the same cell morphology as the strain expressing wild-type *ftsEX* (average length, 5.8 ± 4.1 μM [Table S4]), whereas the strains expressing either *ftsEX*^Δ^*^lp^* or *ftsE^D162N^X*^Δ^*^lp^* contained chains of cells similar to those of the control with the vector (average lengths, 19.4 ± 12.0 and 20.4 ± 9.5 μM, respectively [Table S4]). In stationary phase, the chaining phenotype largely disappeared, in agreement with the notion that cell chaining is due to a delay in cell separation (Fig. S2). These results suggest that under the conditions employed here, the ability of FtsEX to recruit EnvC, but not its ATPase activity, is required for cell separation.

10.1128/mBio.01247-20.2 FIG S2 Morphology of ftsA*^,G366D^ ΔftsEX cells expressing different alleles of *ftsEX* at stationary phase. The experiments used the cells shown in Fig. 5B, except that the cells were allowed to reach stationary phase. Scale bar, 5 μm. Download FIG S2, PDF file, 0.2 MB. Copyright © 2020 Du et al. 2020 Du et al. This content is distributed under the terms of the Creative Commons Attribution 4.0 International license.

10.1128/mBio.01247-20.6 TABLE S4 Lengths of ftsA*^,G366D^ ΔftsEX cells expressing different *ftsEX* alleles in log phase. Download Table S4, DOCX file, 0.01 MB. Copyright © 2020 Du et al. 2020 Du et al. This content is distributed under the terms of the Creative Commons Attribution 4.0 International license.

### ATPase hydrolysis by FtsEX is required for efficient cell separation in the absence of NlpD.

**FIG 6:**
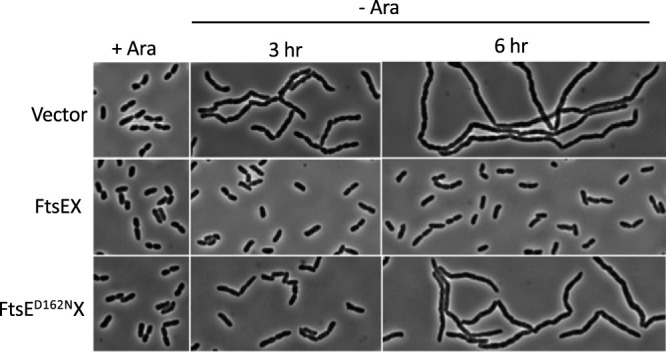
FtsEX contributes to cell separation in the absence of ATP hydrolysis, but it is not sufficient in the absence of the NlpD-mediated cell separation system. SD518 (TB28 *ftsEX*<>*frt nlpD*<>*frt att*^λ^ *P*_BAD_::*ftsEX*) carrying pBS58 (pGB2-*ftsQAZ*) was transformed with derivatives of pEXT22 expressing alleles of *ftsEX* under the control of an IPTG-inducible promoter. The strains were grown in LB with 1.5% NaCl and 0.2% arabinose. At time zero, the cells were collected by centrifugation, washed twice, and resuspended in LB with 1.5% NaCl with or without arabinose. Samples were taken for microscopy at the indicated times after the removal of arabinose. IPTG was not added, since the basal level of expression of *ftsEX* is sufficient for complementation of a *ΔftsEX* mutant.

The results presented above are in contrast to a previous report in which the ATPase activity of FtsEX appeared essential for cell separation (8). However, in that study, *nlpD*, the activator of the other amidase (AmiC), was absent. In addition, increased osmolarity and extra FtsQAZ were provided to suppress the growth defects of the Δ*ftsEX* strain. It is likely that under these conditions, the demand for the cell separation activity conferred by FtsEX is greater. To see if this was the case, we repeated the experiment from the previous report (8) with only a slight modification. We used a *ΔftsEX ΔnlpD* strain containing a copy of *ftsEX* integrated at the lambda attachment site (*att*^λ^) under the control of the arabinose promoter. In the presence of 0.2% arabinose, this strain does not have a cell separation defect, but it displays extensive chaining upon removal of arabinose. We introduced a vector (control) or plasmids harboring either *ftsEX* or *ftsE^D162N^X* under the control of an IPTG-inducible promoter and checked cell morphology upon the removal of arabinose (basal expression from the IPTG-inducible promoter is sufficient for complementation). As shown in Fig. 6, the strain with the vector formed chains by 3 h after the removal of arabinose, and chaining was even more extensive at 6 h. The presence of the *ftsEX* plasmid suppressed chaining, whereas chaining was intermediate between the vector control and the *ftsEX* plasmid when the *ftsE^D162N^X* plasmid was present (Table S5). These results are consistent with the previous report and suggest that deletion of *nlpD* indeed reveals a requirement for the ATPase activity of FtsEX in cell separation.

10.1128/mBio.01247-20.7 TABLE S5 Lengths of SD518 (*ftsA ΔftsEX ΔnlpD att^λ^ P*_BAD_::*ftsEX/*pSC101 *ftsQAZ*) cells expressing different *ftsEX* alleles after depletion of arabinose. Download Table S5, DOCX file, 0.01 MB. Copyright © 2020 Du et al. 2020 Du et al. This content is distributed under the terms of the Creative Commons Attribution 4.0 International license.

The *ΔftsEX ΔnlpD att*^λ^::*P*_BAD_::*ftsEX* strain used in the experiment presented above is still sensitive to the division-inhibitory activity of FtsE^D162N^X. However, the inhibitory activity is likely suppressed by the extra FtsQAZ, which may confound the interpretation of the results. Therefore, we reexamined the requirement for the ATPase activity of FtsEX for cell separation in the absence of NlpD by using the ftsA*^,^*^G366D^* allele described above, which is resistant to the division-inhibitory activity of *ftsE^D162N^X* and suppresses the division defect of the Δ*ftsEX* mutant. We generated two derivatives of the Δ*ftsEX* Δ*nlpD att*^λ^::*P*_BAD_::*ftsEX* strain, differing only in their *ftsA* alleles (ftsA* or ftsA*^,^*^G366D^*). In the absence of arabinose, these two strains underwent extensive cell chaining, since both cell separation systems were inactive. We then introduced a vector or plasmids expressing *ftsEX* or *ftsE^D162N^X* under the control of an IPTG-inducible promoter to examine the role of the ATPase activity.

**FIG 7:**
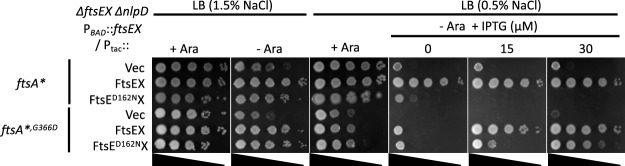
Effects of the FtsA-FtsEX interaction and ATP hydrolysis by FtsEX on its ability to rescue a *ΔftsEX ΔnlpD* strain. A *ΔftsEX ΔnlpD P*_BAD_::*ftsEX* strain with either ftsA* or ftsA*^,^*^G366D^* was transformed with a vector or plasmids expressing alleles of *ftsEX* under the control of an IPTG-inducible promoter. The transformants were spotted onto LB plates with or without arabinose and different concentrations of NaCl and IPTG. Plates were incubated overnight at 37°C and photographed. The strains were SD523 (TB28 ftsEX<>frt nlpD<>frt ftsA* att^λ^ [*P*_BAD_::*ftsEX*]) and SD524 (TB28 ftsEX<>frt nlpD<>frt ftsA*^,^*^G366D^ att*^λ^ [*P*_BAD_::*ftsEX*]) carrying plasmids expressing WT *ftsEX* (pSD221 [pEXT22 *P*_tac_::*ftsEX*]), *ftsE^D162N^X* (pSD221-D162N [pEXT22 *P*_tac_::*ftsE^D162N^X*]), or the vector pEXT22.

On LB plates with 1.5% NaCl, which prevents the cell lysis that accompanies extensive cell chaining, these two strains with the vector grew regardless of the presence of arabinose (Fig. 7). However, on LB plates with 0.5% NaCl, neither strain grew in the absence of arabinose, whereas in the presence of arabinose, the strain with the ftsA*^,^*^G366D^* allele grew less well than the strain with the ftsA* allele. The growth defect of these two strains on LB plates with 0.5% NaCl allowed us to do complementation tests. As shown in Fig. 7, the presence of *ftsEX* on the plasmid rescued the growth of both strains, but the strain with ftsA*^,^*^G366D^* required more IPTG, indicating that *ftsEX* was less efficient in this strain than in the ftsA* strain. As expected, the plasmid with *ftsE^D162N^X* was unable to complement the Δ*ftsEX* Δ*nlpD* strain with the ftsA* mutation, since this strain is sensitive to its inhibitory activity. However, *ftsE^D162N^X* complemented the ftsA*^,^*^G366D^* strain, where it cannot inhibit division, although a higher level of IPTG was required than when *ftsEX* was on the plasmid. These results suggest that *ftsE^D162N^X* can promote cell separation but is less effective than *ftsEX*.

**FIG 8:**
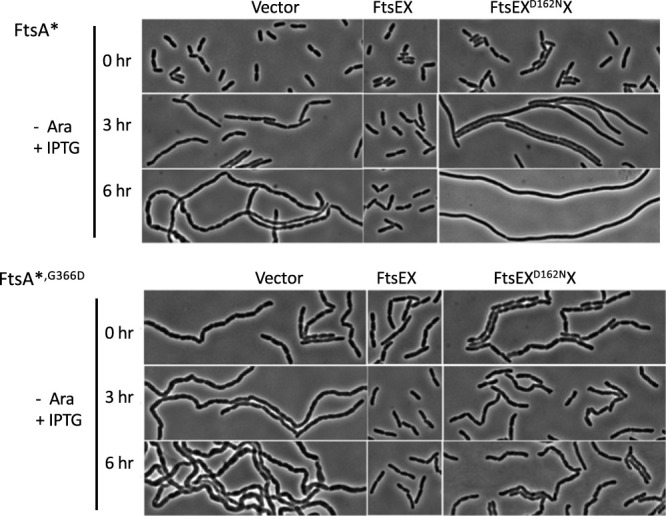
Effect of the FtsA-FtsEX interaction and ATP hydrolysis by FtsEX on its ability to promote cell separation in the absence of NlpD. The strains from Fig. 7 were grown in LB with 1.5% NaCl and 0.2% arabinose. At time zero, the cells were collected by centrifugation, washed, and resuspended in LB without arabinose but containing 1.5% NaCl and 30 μM IPTG. At the indicated times, samples were taken for photography. Note that the strain with the ftsA*^,^*^G366D^* allele containing the vector is chaining at time zero, even though it was grown in the presence of arabinose to induce *ftsEX* from its ectopic location on the chromosome.

We set out to examine the effect of *ftsEX* or *ftsE^D162N^X* on cell chaining; however, in LB with 0.5% NaCl, both strains with the vector lysed before the chaining phenotype was evident. Therefore, we examined the chaining phenotypes of these two strains in 1.5% NaCl. As shown in Fig. 8, the *ΔnlpD att*^λ^::*P*_BAD_::*ftsEX* strain with the ftsA* mutation and the vector displayed a normal morphology at time zero (with arabinose removed and 30 μM IPTG added), but cell chaining became evident after 3 h and was extensive by 6 h (Fig. 8; Table S6). Induction of *ftsEX* from the plasmid prevented the chaining phenotype, whereas induction of *ftsE^D162N^X* from the plasmid resulted in inhibition of division and smooth filamentation, as expected. Surprisingly, cells from the strain with the vector and the ftsA*^,G366D^ allele were already chaining at time zero (Fig. 8; Table S6). This observation revealed that *ftsEX* induced from chromosomal *att*^λ^::*P*_BAD_::*ftsEX* was not sufficient to suppress the chaining, indicating that *ftsEX* was less effective in the presence of ftsA*^,^*^G366D^*, in agreement with the complementation results (Fig. 7). Upon the removal of arabinose, the chaining of cells with the vector became more extensive, whereas induction of *ftsEX* from the plasmid decreased the chain length by 6 h, indicating that the higher level of FtsEX partially overcame the loss of interaction with FtsA (Fig. 8; Table S6). Induction of *ftsE^D162N^X* from the plasmid also prevented the severe chaining seen with the vector, but it was not as effective as *ftsEX* (Fig. 8; Table S6). Taken together, these results demonstrate that the ATPase mutant of FtsEX promotes cell separation but is not sufficient to produce a WT phenotype when the partially redundant NlpD system is inactivated. Also, *ftsEX* is not as efficient at promoting cell separation when it cannot interact with *ftsA* (due to the *ftsA^G366D^* mutation).

10.1128/mBio.01247-20.8 TABLE S6 Lengths of cells of strain SD523 (ftsA* ΔftsEX ΔnlpD att^λ^ P_BAD_::*ftsEX*) and SD524 (ftsA*^,G366D^ ΔftsEX ΔnlpD att^λ^ P_BAD_::*ftsEX*) expressing different *ftsEX* alleles after the depletion of arabinose. Download Table S6, DOCX file, 0.01 MB. Copyright © 2020 Du et al. 2020 Du et al. This content is distributed under the terms of the Creative Commons Attribution 4.0 International license.

### The FtsX-FtsA interaction is important for efficient cell separation in the absence of NlpD.

The results presented above (Fig. 7) suggested that in the absence of the FtsA-FtsX interaction, the efficiency of the FtsEX-mediated cell separation activity was reduced. First, a higher level of FtsEX (i.e., more IPTG) was required for the Δ*ftsEX* Δ*nlpD att*^λ^::*P*_BAD_::*ftsEX* strain to grow when the ftsA*^,^*^G366D^* allele was present than when the ftsA* allele was present (in the absence of arabinose on LB plates with 0.5% NaCl). Also, when ftsA*^,^*^G366D^* was present, cells displayed a strong cell chaining phenotype at time zero, even though arabinose was present to induce *ftsEX* from the integrated copy (Fig. 8). In contrast, the Δ*ftsEX* Δ*nlpD* strain with ftsA* did not have a chaining phenotype. The only difference between these two strains is the *ftsA^G366D^* mutation. The fact that this mutation abolishes the FtsEX-FtsA interaction (17) suggests that this interaction is important for efficient cell separation under the conditions tested. However, in the rifampin sensitivity test (Fig. 5), there was no difference between the strains containing ftsA* and ftsA*^,^*^G366D^*; in that case, though, *nlpD* was present, and *ftsEX* was expressed at a high level from a plasmid.

**FIG 9:**
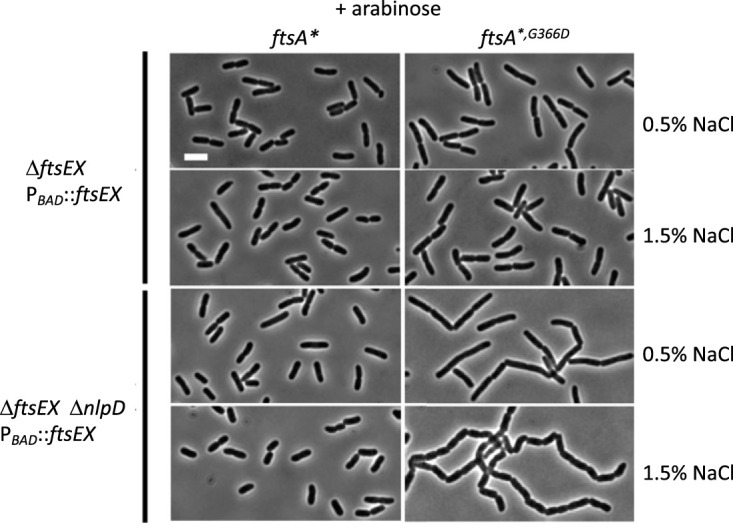
Loss of the FtsEX-FtsA interaction delays cell division and cell separation. The effect of FtsEX-FtsA interaction on cell length and cell separation was assessed in strains with ftsA* or ftsA*^,^*^G366D^* on the chromosome and with or without *nlpD*. The strains were grown in LB with 0.2% arabinose and either 0.5% or 1.5% NaCl. The strains were SD528 [TB28 ftsA* leu::Tn*10 ftsEX*<>*frt att^λ^* (*P*_BAD_::*ftsEX*)], SD529 [TB28 ftsA*^,^*^G366D^ leu*::Tn*10 ftsEX*<>*frt att^λ^* (*P*_BAD_::*ftsEX*)], SD523 [TB28 *nlpD*<>*frt leu*::Tn10 ftsA* ftsEX<>*frt att^λ^* (*P*_BAD_::*ftsEX*)], and SD524 [TB28 *nlpD*<>*frt leu*::Tn10 ftsA*^G366D^ ftsEX<>*frt att^λ^* (*P*_BAD_::*ftsEX*)].

To examine the effect of the FtsA-FtsX interaction on cell division more carefully, we compared the cell length and chaining phenotype of ΔftsEX ftsA* cells to those of ΔftsEX ftsA*^,^*^G366D^* cells ectopically expressing *ftsEX* from a chromosomal copy under the control of an arabinose-inducible promoter, with or without NlpD. In the presence of arabinose (LB with 0.5% NaCl), the strain with ftsA* had an average cell length of 3.8 ± 0.88 μm, whereas the strain with ftsA*^,^*^G366D^* had an average cell length of 5.2 ± 1.25 μm (Table S7), indicating that loss of the interaction between FtsEX and FtsA delays cell division. Also, the failure to see a difference earlier (Fig. 4) was likely due to the higher level of expression of *ftsEX* from a plasmid. Deletion of *nlpD* had little effect on the average cell length of the ΔftsEX ftsA* strain; however, the average cell length of the ΔftsEX ftsA*^,G366D^ strain doubled to 10.9 ± 5.7 μm (Fig. 9; Table S7). Increasing NaCl to 1.5% had little effect on cell separation in the ΔftsEX ftsA* strain but exacerbated the defect of the ΔftsEX ftsA*^,G366D^ strain, for which cell length was further increased and chaining was more pronounced. These results confirmed that loss of the FtsEX-FtsA interaction leads to a delay in cell division, which becomes more evident in the absence of *nlpD* and is further exacerbated by increased salt concentrations.

10.1128/mBio.01247-20.9 TABLE S7 Average cell lengths of ftsA* and ftsA*^,G366D^ strains with or without *nlpD*. Download Table S7, DOCX file, 0.01 MB. Copyright © 2020 Du et al. 2020 Du et al. This content is distributed under the terms of the Creative Commons Attribution 4.0 International license.

## DISCUSSION

In this study, we continued to explore the involvement of FtsEX in cell division, and we report several new findings that highlight its regulatory role. First, we find that an FtsEX mutant unable to hydrolyze ATP blocks cells in the process of constriction without disrupting the septal ring, implying that ATP hydrolysis by FtsEX is required throughout septation. Thus, the ATPase mutant of FtsEX mimics the action of cephalexin, a well-characterized inhibitor of FtsI (PBP3). Second, we found that a *ΔftsEX* strain is more sensitive to a hydrophobic antibiotic than a *ΔenvC* strain, a finding consistent with roles of FtsEX in more than cell separation. Third, we observed that in the absence of ATP hydrolysis, FtsEX still promotes cell separation. This activity is sufficient to promote cell separation when an overlapping separation pathway (*nlpD*) is present but insufficient if this pathway is missing. Last, we found that the interaction of FtsEX with FtsA is required for efficient cell division and cell separation. This effect is observed when *nlpD* is present but is more pronounced when *nlpD* is removed. These results argue that a physical coupling of the cell separation system to the septal PG synthesis machinery via the FtsEX-FtsA interaction is important for normal cell division and cell separation.

In our previous work (17), we found that an ATPase mutant of FtsEX blocked the start of constriction by acting on FtsA. Here, we extend those findings and show that FtsE^D162N^X also blocks ongoing septation and thus phenocopies cephalexin. This means that FtsEX must continually hydrolyze ATP throughout the constriction process. Constriction is initiated when FtsN arrives, which is thought to switch FtsA in the cytoplasm and FtsQLB in the periplasm to the “on” state, leading to activation of FtsWI (25–27). In this model, FtsE^D162N^X could block division by preventing FtsA from reaching the on state or communicating with downstream proteins (17). Recent evidence indicates that the septal PG synthase FtsW exists in two types of processive moving complexes at the Z ring (32). The faster complex is inactive and is driven by FtsZ treadmilling, whereas the slower-moving one likely represents the active complex synthesizing septal PG. This switch from a fast-moving to a slow-moving complex appears to correlate with the activation of the complex by FtsN. In this scenario, the ATPase mutant of FtsEX may prevent the switch, but the mechanism remains to be determined.

Cell separation in E. coli employs two distinct systems involving three amidases and two activators (18, 23). FtsEX regulates AmiA and AmiB through EnvC, whereas NlpD activates the third amidase, AmiC. Although it is generally thought that the ATPase activity of FtsEX is required for the activation of its cognate amidases, *in vitro* assays have raised some doubt (18). In these assays, full-length EnvC stimulated amidase activity *in vitro* to the same extent as the C-terminal fragment of EnvC, and FtsEX was not required (18). It is difficult, however, to tease out the role of FtsEX ATPase activity *in vivo*, because it is also necessary for septal PG synthesis (17). Additional confounding issues include the ability of the FtsEX ATPase mutant to inhibit division and the partially redundant pathways to activate amidases.

To circumvent these issues, we took advantage of the ftsA*^,^*^G366D^* allele, which prevents the interaction of FtsEX with FtsA (hence rendering cells resistant to FtsE^D162N^X) and also bypasses the requirement of FtsEX for septal PG synthesis (17). This mutant uncouples the essential role of FtsEX in septal PG synthesis from its role in septal PG hydrolysis, allowing us to assess the contribution of ATP hydrolysis by FtsEX to cell separation. FtsE^D162N^X suppressed the increased sensitivity of *ftsEX* cells to the hydrophobic drug rifampin in an EnvC-dependent manner. Importantly, this rescue was dependent on EnvC and also eliminated the chaining morphology, indicating that FtsE^D162N^X was still able to promote cell separation. However, when NlpD was deleted, the cell chaining was only partially suppressed (Fig. 6 to 8). This is consistent with the previous report which found that cell chains were somewhat shorter with the ATPase mutant than with the vector control (8). These observations suggest that EnvC can activate AmiA and AmiB to some extent when it is recruited to the septum by FtsEX; however, ATP hydrolysis by FtsEX is probably required for optimal activity of this cell separation system. It should be noted that based on the analogy to MacB (12), FtsE^D162N^ is locked in the ATP-bound state. If this is the active form of FtsEX (for activating amidases), it may contribute to cell separation but is less efficient than the WT due to a lack of dynamics.

Using the ftsA*^,^*^G366D^* mutation, we also uncovered an unexpected role for the FtsEX-FtsA interaction in cell separation. This role emerged from the unexpected observation that a higher level of *ftsEX* was required to complement the *ΔftsEX ΔnlpD* strain in the presence of the ftsA*^,^*^G366D^* mutation than in the presence of ftsA* (Fig. 7). Also, *ΔftsEX ΔnlpD* cells with the ftsA*^,^*^G366D^* mutation displayed a chaining phenotype even when FtsEX was provided at a level that promotes cell separation efficiently in *ΔftsEX ΔnlpD* cells with the ftsA* mutation (Fig. 8). These results indicate that cell separation is less effective when the FtsEX-EnvC-amidase cell separation system is uncoupled from FtsA, which is likely associated with the active FtsWI complex. This defect in cell separation is further amplified in the absence of the other cell separation system. It is likely that uncoupling the FtsEX-mediated cell separation system from the septal PG synthesis machinery delays septal PG hydrolysis, leading to cell chaining. A delay in the production of denuded PG strands by the amidases would reduce the accumulation at the septum of SPOR domain proteins (including FtsN and DedD) that bind the denuded glycan strands (33, 34). Decreased accumulation of these proteins would decrease the activation of FtsWI, leading to a decreased rate of septal PG synthesis and increased cell length.

Based on previous findings (8, 16, 17) and our observations here, we propose a model for how FtsEX coordinates septal PG synthesis and hydrolysis (Fig. 1A). FtsEX localizes to the Z ring via an interaction between FtsE and the conserved C-terminal peptide (CCTP) of FtsZ (16). Once at the Z ring, FtsX interacts with FtsA, promoting divisome assembly, and recruits the amidase activator EnvC through its periplasmic loop. This recruitment phase does not require ATP hydrolysis by FtsEX but acts to link the septal PG synthesis machinery (FtsA-FtsQLB-FtsWI) and the septal PG hydrolysis machinery (FtsA-FtsEX-EnvC-AmiA/AmiB) (Fig. 1A). The arrival of FtsN switches FtsA and FtsQLB to the on state, activating FtsWI to synthesize septal PG. FtsEX must hydrolyze ATP at this step, or septal PG synthesis will be blocked. Once new septal PG is synthesized, amidases are activated by EnvC to cleave the stem peptide, leading to timely cell separation (Fig. 1A). In the absence of ATP hydrolysis, FtsEX is able to recruit EnvC and activate AmiA and AmiB, sufficiently to promote cell separation when the overlapping cell separation system controlled by NlpD is present. However, in its absence, this activity is insufficient, indicating a role for ATP hydrolysis. In the absence of the FtsEX-FtsA interaction (due to the *ftsA^G366D^* mutation), FtsEX still localizes at the Z ring via an interaction with FtsZ. However, the FtsEX-EnvC-amidase cell separation system is uncoupled from the FtsA-FtsQLB-FtsWI septal PG synthesis machinery (Fig. 1B), resulting in a delay in the production of denuded peptidoglycan strands, which, in turn, delays the accumulation of SPOR domain proteins and leads to a delay in cell division. This model will be useful for further study of the role of FtsEX in regulating cell division and cell wall hydrolysis in E. coli and other bacterial species.

## MATERIALS AND METHODS

### Media, bacterial strains, plasmids, and growth conditions.

Cells were grown in LB alone or LB plus 0.2 M sucrose medium (1% tryptone, 0.5% yeast extract, 0.5% or 1.5% NaCl, and 0.05 g/liter thymine) at 30°C or 37°C. When needed, antibiotics were used at the following concentrations: ampicillin , 100 μg/ml; spectinomycin , 25 μg/ml; kanamycin , 25 μg/ml; tetracycline , 25 μg/ml; chloramphenicol ,  20 μg/ml. Rifampin was used at the concentrations indicated in the figures for the drug sensitivity test. The strains and plasmids used in this study are listed in Tables 1 and 2, respectively.

**TABLE 1:** Bacterial strains used in this study

Strain	Genotype	Source or reference
JS238	MC1061 *araD* Δ(*ara leu*) *galU galK hsdS rpsL* Δ(*lacIOPZYA*)*X74 malP*::*lacIQ srlC*::Tn*10 recA1*	Lab collection
JW2712	BW25113 *nlpD*::*kan*	37
HC261	TB28 *zapA-GFP* Cam^r^	38
PS2343	W3110 *leu*::Tn10 ftsA*	39
S3	W3110 *leu*::Tn*10*	40
SD208	W3110 *envC*::*kan*	This study
SD220	W3110 *leu*::Tn*10 ftsEX*::*cat*	17
SD221	W3110 *leu*::Tn10 ftsA* ftsEX::*cat*	17
SD249	W3110 *leu*::Tn10 ftsA*^,G366D^	17
SD262	W3110 *leu*::Tn10 ftsA*^,G366D^ ftsEX::*cat*	17
SD516	TB28 *ftsEX<>frt att^λ^* (*P*_BAD_::*ftsEX*) *nlpD*::*kan*	This study
SD518	TB28 *ftsEX<>frt att^λ^* (*P*_BAD_::*ftsEX*) *nlpD<>frt*	This study
SD523	TB28 *ftsEX<>frt att^λ^* (*P*_BAD_::*ftsEX*) *nlpD<>frt leu*::Tn10 ftsA*	This study
SD524	TB28 *ftsEX<>frt att^λ^* (*P*_BAD_::*ftsEX*) *nlpD<>frt leu*::Tn10 ftsA*^,G366D^	This study
SD528	TB28 *ftsEX<>frt att^λ^* (*P*_BAD_::*ftsEX*) *leu*::Tn10 ftsA*	This study
SD529	TB28 *ftsEX<>frt att^λ^* (*P*_BAD_::*ftsEX*) *leu*::Tn10 ftsA*^,G366D^	This study
TB35	TB28 *envC*::*kan*	41
TU191	TB28 *ftsEX<>frt att^λ^* (*P*_BAD_::*ftsEX*)	8

**TABLE 2:** Plasmids used in this study

Plasmid	Genotype	Source or reference
pBS58	pSC101 *aadA ftsQAZ*	42
pCP20	pSC101(Ts) *aadA repA*(Ts) *P*_BAD_::*flp*	43
pDSW208	pDSW208 *bla P*_trc_::*gfp*	44
pSD213	pDSW208 *bla P*_trc_::*ftsEX^Δlp^*	This study
pSD213-D162N	pDSW208 *bla P*_trc_::*ftsE^D162N^X^Δlp^*	This study
pSD221	pEXT22 *kan P*_tac_::*ftsEX*	17
pSD221-D162N	pEXT22 *kan P*_tac_::*ftsE^D162N^X*	17
pSEB428	pDSW208 *bla P*_trc_::*ftsEX*	45
pSEB428-D162N	pDSW208 *bla P*_trc_::*ftsE^D162N^X*	17

### Construction of strains.

Strains were constructed largely by P1-mediated transduction. Strain SD208 (W3110 *envC*::*kan*) was constructed by the introduction of the *envC*::*kan* allele from strain TB35 (TB28 *envC*::*kan*) into W3110. Transductants were selected on LB plates with kanamycin at 30°C. Strain SD516 [TB28 *ftsEX<>frt att^λ^* (*P*_BAD_::*ftsEX*) *nlpD*::*kan*] was constructed by P1-mediated transduction of the *nlpD*::*kan* cassette from strain JW2712 (BW25113 *nlpD*::*kan*) into strain TU191 [TB28 *ftsEX<>frt att^λ^* (*P*_BAD_::*ftsEX*)]. Transductants were selected on LB medium with 1.5% NaCl, 0.2% arabinose, and kanamycin. The *kan* cassette was removed from *nlpD*::*kan* strains by using plasmid pCP20 to create strain SD518 [TB28 *ftsEX<>frt att^λ^* (*P*_BAD_::*ftsEX*) *nlpD<>frt*]. Strains SD523 [TB28 *ftsEX<>frt att^λ^* (*P*_BAD_::*ftsEX*) *nlpD<>frt leu*::Tn10 ftsA*] and SD524 [TB28 *ftsEX<>frt att^λ^* (*P*_BAD_::*ftsEX*) *nlpD<>frt leu*::Tn10 ftsA*^,^*^G366D^*] were constructed by P1-mediated transduction of *leu*::Tn10 ftsA* or *leu*::Tn10 ftsA*^,^*^G366D^* from strain PS2343 (*leu*::Tn10 ftsA*) or strain SD249 (*leu*::Tn10 ftsA*^,^*^G366D^*) into strain SD518. ftsA* refers to the *ftsA^R286W^* allele (35). Transductants were selected on LB medium with 1.5% NaCl, 0.2% arabinose, and tetracycline. The *ftsA* coding sequences from at least four transductants were PCR amplified and sequenced, and those with the ftsA* or ftsA*^,^*^G366D^* mutation were saved. Strain SD528 [TB28 *ftsEX<>frt att^λ^* (*P*_BAD_::*ftsEX*) *leu*::Tn10 ftsA*] and SD529 were constructed similarly by P1-mediated transduction of *leu*::Tn10 ftsA* or *leu*::Tn10 ftsA*^,^*^G366D^* into strain TU191 [TB28 *ftsEX<>frt att^λ^* (*P*_BAD_::*ftsEX*)].

### Determination of the effect of FtsE^D162N^X overexpression or cephalexin on cell constriction, contraction of Z rings, and septal PG synthesis.

To follow cell constriction and Z ring contraction, an overnight culture of HC261/pSD221-D162N (pEXT22 *P*_tac_::*ftsE^D162N^X*) was diluted 100-fold in LB with sucrose and antibiotics and was grown at 30°C. After 2 h, 2 μl of the culture was spotted onto a 2% agarose pad containing LB plus sucrose. ZapA-GFP and cell division were followed for 40 min. To follow cell constriction and Z ring contractions in cells expressing FtsE^D162N^X, an overnight culture of HC261/pSD221-D162N was diluted 100-fold in LB with sucrose and antibiotics and was grown at 30°C until the optical density at 600 nm (OD_600_) reached about 0.6. The culture was diluted 5-fold in the same medium containing 250 μM IPTG. After induction for 1 h, 2 μl of the culture was spotted onto a 2% agarose pad containing LB plus sucrose with IPTG and was monitored for 45 min. To compare the effects of FtsE^D162N^X overexpression and cephalexin on cell division, HC261/pSD221-D162N cells were cultured as described above, and IPTG (250 μM) or cephalexin (20 μg/ml) was added. After 1 h or 45 min, respectively, cells were fixed with paraformaldehyde and glutaraldehyde and were immobilized on 2% agarose for fluorescence microscopy. 4′,6-Diamidino-2-phenylindole (DAPI) was added at a final concentration of 200 ng/ml 5 min before fixation to monitor the distribution of nucleoids.

To check the effect of FtsE^D162N^X overexpression or cephalexin on HADA incorporation (septal PG synthesis), HC261/pSD221-D162N (pEXT22 *P*_tac_::*ftsE^D162N^X*) cells were cultured and treated as described above. A 200-μl sample was then taken from each culture and incubated with 2 μl of HADA in dimethyl sulfoxide (DMSO; final concentration, 0.25 mM) for 1 min. After the incubation, the cells were immediately fixed with paraformaldehyde and glutaraldehyde for 15 min on ice and were then washed four times with phosphate-buffered saline (PBS). The cells were then resuspended in 50 μl of PBS and were spotted onto an agarose pad for imaging. A sample from the culture with IPTG was also taken at 90 min and was treated similarly.

### Measurement of cell lengths.

To measure the length of *ΔenvC ΔftsEX* cells with or without the *ftsA* mutations (Table S3), overnight cultures were diluted 1:100 in LB with 0.2 M sucrose and were grown at 30°C. After 3 h, cells were fixed with paraformaldehyde and glutaraldehyde, immobilized on a 2% agarose pad, and photographed. Cell length was measured using MetaMorph software.

The lengths of ftsA*^,^*^G366D^ ΔftsEX* cells expressing different *ftsEX* alleles (Table S4) were measured as described above for Table S3. To measure the lengths of strain SD518 [TB28 *ftsEX*<>*frt nlpD*<>*att*^λ^::*P*_BAD_::*ftsEX*/pBS58 (pGB2-*ftsQAZ*)] cells expressing different alleles of *ftsEX* (Table S5), overnight cultures were diluted 1:100 in LB with 1.5% NaCl and 0.2% arabinose and were grown for 3 h at 30°C. At time zero, the cells were collected by centrifugation, washed twice, and resuspended in LB with 1.5% NaCl with or without arabinose. Samples were taken for microscopy at various times after the removal of arabinose. Cells were detected and measured using the ImageJ plug-in MicrobeJ (36).

The lengths of cells from strains SD523 (ftsA* ΔftsEX ΔnlpD att^λ^ P_BAD_::*ftsEX*) and SD524 (ftsA*^,^*^G366D^ ΔftsEX ΔnlpD att^λ^ P*_BAD_::*ftsEX*) expressing different *ftsEX* alleles (Table S6) were measured similarly to the lengths of strain SD518 cells, except that 30 μM IPTG was added to the culture.

Overnight cultures of SD528 [TB28 ftsA* leu::Tn*10 ftsEX*<>*frt att^λ^* (*P*_BAD_::*ftsEX*)], SD529 [TB28 ftsA*^,^*^G366D^ leu*::Tn*10 ftsEX*<>*frt att^λ^* (*P*_BAD_::*ftsEX*)], SD523 [TB28 *nlpD*<>*frt leu*::Tn10 ftsA* ftsEX<>*frt att^λ^* (*P*_BAD_::*ftsEX*)], and SD524 [TB28 *nlpD*<>*frt leu*::Tn10 ftsA*^,G366D^ ftsEX<>*frt att^λ^* (*P*_BAD_::*ftsEX*)] were diluted 1:100 in in LB with either 0.5% or 1.5% NaCl and 0.2% arabinose and were grown at 30°C. After 3 h, cells were fixed with paraformaldehyde and glutaraldehyde, immobilized on a 2% agarose pad, and photographed. Cell length was measured using MetaMorph software.

### Rifampin sensitivity test.

To determine the sensitivities of various strains to rifampin, overnight cultures were serially diluted 10-fold, and 3 μl was spotted onto LB-plus-sucrose plates with or without increasing concentrations of rifampin. The plates were incubated at 37°C overnight and photographed.

To determine whether FtsEX mutants can correct the sensitivity to rifampin, pDSW208 (*gfp*), pSEB428 (*ftsEX*), and their derivatives were transformed into strain SD262 (*ftsEX*::cat ftsA*^,^*^G366D^*) and transformants selected on LB-plus-sucrose plates with ampicillin. The transformants were then restreaked onto LB-plus-sucrose plates with antibiotics, with or without 4 μg/ml rifampin. The plates were incubated at 37°C overnight and photographed.
