# Supplementary figures and images for: Roles of ATP Hydrolysis by FtsEX and Interaction with FtsA in Regulation of Septal Peptidoglycan Synthesis and Hydrolysis
Source: mBio. 2020 Jul 7;11(4):e01247-20. doi: 10.1128/mBio.01247-20 (PMC7343993; doi:10.1128/mBio.01247-20)

Fig. S1

- IPTG

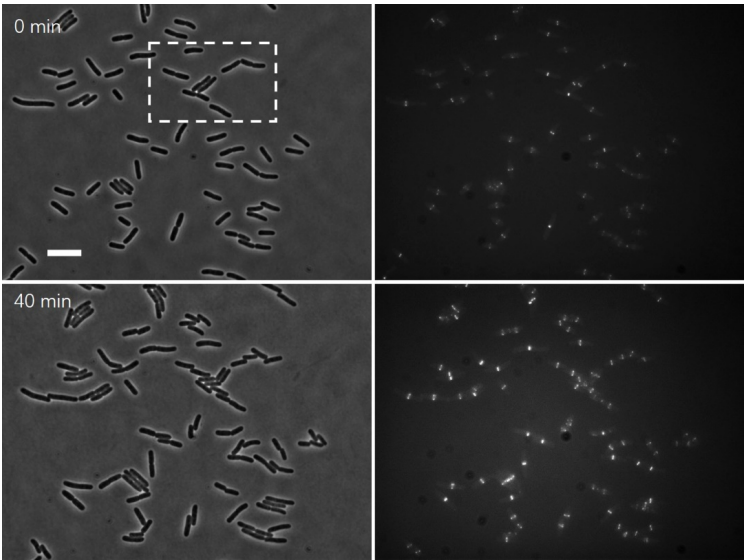

+ IPTG

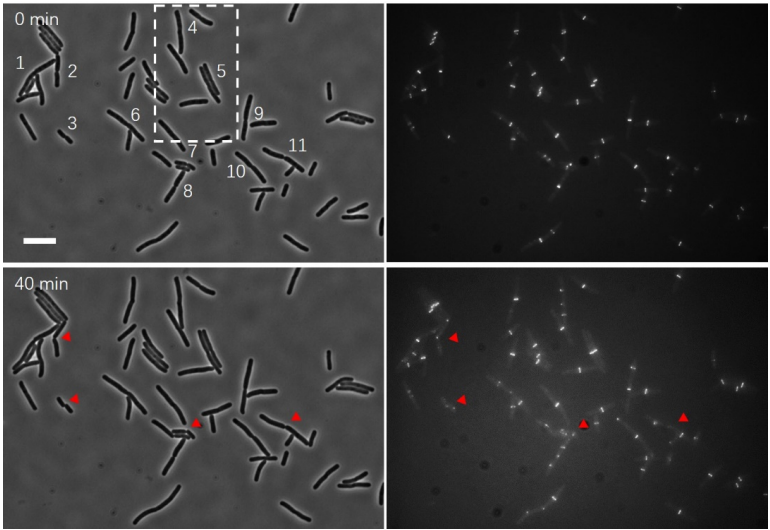

Supplement: FIG S1 [file mBio.01247-20-sf001.pdf]

Fig. S2

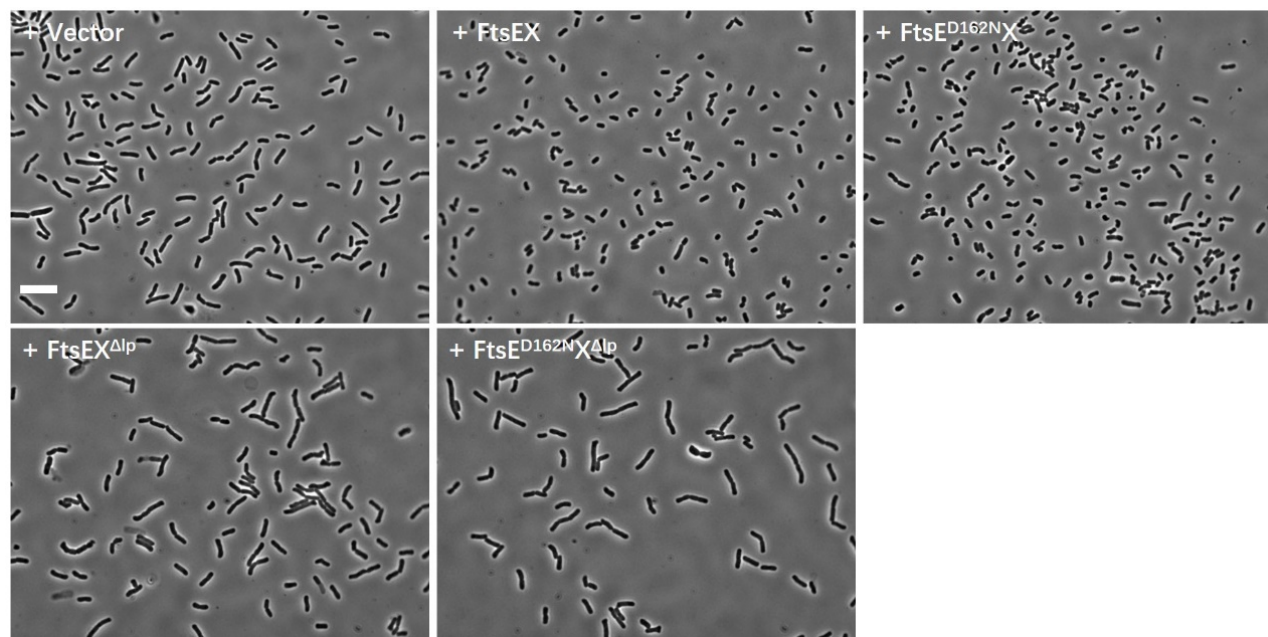

Supplement: FIG S2 [file mBio.01247-20-sf002.pdf]
