# Supplementary material for: Roles of ATP Hydrolysis by FtsEX and Interaction with FtsA in Regulation of Septal Peptidoglycan Synthesis and Hydrolysis
Source: mBio. 2020 Jul 7;11(4):e01247-20. doi: 10.1128/mBio.01247-20 (PMC7343993; doi:10.1128/mBio.01247-20)
Supplement: TABLE S1 [file mBio.01247-20-st001.docx]

**Table S1. Z ring formation and cell constriction in cells with or without FtsE^D162N^X overproduction.**

|  | **0 min** | | | | **40 min** | | | |
| --- | --- | --- | --- | --- | --- | --- | --- | --- |
| **IPTG** | **# cells** | **# Z rings** | **# constricting cells** | **# Z rings associated with a constriction** | **# cells** | **# Finished constriction^a^** | **# Z rings** | **# constricting cells** |
| - | 56 | 49 | 18 | 18 | 82 | 18 | 79 | 26 |
| + | 49 | 46 | 11 | 11 | 53 | 4 | 46 | 7 |

**^a^** Constrictions that were visible at time 0 were completed by the end of the 40 minute observation period.
