# Supplementary material for: Roles of ATP Hydrolysis by FtsEX and Interaction with FtsA in Regulation of Septal Peptidoglycan Synthesis and Hydrolysis
Source: mBio. 2020 Jul 7;11(4):e01247-20. doi: 10.1128/mBio.01247-20 (PMC7343993; doi:10.1128/mBio.01247-20)
Supplement: TABLE S2 [file mBio.01247-20-st002.docx]

**Table S2. Co-localization of Z rings and HADA in cells treated with cephalexin or overexpressing FtsE^D162N^X.**

| **Time**  **(min)** | **Treatment** | **# cells** | **# Z rings** | **# HADA rings** | **% co-localization** |
| --- | --- | --- | --- | --- | --- |
| 0 | None | 194 | 142 | 54 | 38 |
| 45 | Cephalexin | 179 | 154 | 0 | 0 |
| 45 | FtsE^D162N^X | 206 | 152 | 14 | 9.2 |
| 90 | FtsE^D162N^X | 154 | 180 | 10 | 5.5 |
