# Supplementary material for: Roles of ATP Hydrolysis by FtsEX and Interaction with FtsA in Regulation of Septal Peptidoglycan Synthesis and Hydrolysis
Source: mBio. 2020 Jul 7;11(4):e01247-20. doi: 10.1128/mBio.01247-20 (PMC7343993; doi:10.1128/mBio.01247-20)
Supplement: TABLE S3 [file mBio.01247-20-st003.docx]

**Table S3. Average cell lengths of *ftsEX* and *envC* deletion strains.**

| **Strain** | **# Cells** | **Average length ^a^ ± STDEV (μm)** |
| --- | --- | --- |
| *ΔenvC* | 263 | 8.3 ± 3.8 |
| *ΔftsEX* | 95 | 12.6 ± 10.5 |
| *ΔftsEX ftsA** | 161 | 6.1 ± 2.9 |
| *ΔftsEX ftsA*^,G366D^* | 206 | 6.6 ± 2.9 |

^a^ The average length refers to the length of cell chains.
