# Supplementary material for: Roles of ATP Hydrolysis by FtsEX and Interaction with FtsA in Regulation of Septal Peptidoglycan Synthesis and Hydrolysis
Source: mBio. 2020 Jul 7;11(4):e01247-20. doi: 10.1128/mBio.01247-20 (PMC7343993; doi:10.1128/mBio.01247-20)
Supplement: TABLE S4 [file mBio.01247-20-st004.docx]

**Table S4. Length of *ftsA*^,G366D^ ΔftsEX* cells expressing different *ftsEX* alleles in log phase.**

| ***ftsEX* alleles** | **# Cells** | **Average length ^a^ ± STDEV (μm)** |
| --- | --- | --- |
| - | 20 | 21.1 ± 9.3 |
| *ftsEX* | 126 | 5.7 ± 3.8 |
| *ftsE^D162N^X* | 79 | 5.8 ± 4.1 |
| *ftsEX^Δlp^* | 18 | 19.4 ± 12.0 |
| *ftsE^D162N^X^Δlp^* | 24 | 20.4 ± 9.5 |

^a^ The average length refers to the length of cell chains.
