# Supplementary material for: Roles of ATP Hydrolysis by FtsEX and Interaction with FtsA in Regulation of Septal Peptidoglycan Synthesis and Hydrolysis
Source: mBio. 2020 Jul 7;11(4):e01247-20. doi: 10.1128/mBio.01247-20 (PMC7343993; doi:10.1128/mBio.01247-20)
Supplement: TABLE S5 [file mBio.01247-20-st005.docx]

**Table S5. Length of SD518 (*ftsA, ΔftsEX ΔnlpD att^λ^P_BAD_::ftsEX /* pSC101*, ftsQAZ*) cells expressing different *ftsEX* alleles after depletion of arabinose.**

| **Arabinose depletion time (h)** | ***ftsEX* allele** | **# Cells** | **Average length ^a^ ± STDEV (μm)** |
| --- | --- | --- | --- |
| 0 | - | 281 | 2.8 ± 0.8 |
|  | ftsEX | 283 | 3.0 ± 0.9 |
|  | *ftsE^D162N^X* | 267 | 3.2 ± 1.0 |
| 3 | - | 107 | 10.7 ± 4.4 |
|  | ftsEX | 233 | 3.0 ± 1.1 |
|  | *ftsE^D162N^X* | 151 | 5.6 ± 2.3 |
| 6 | - | 10 | 82.6 ± 44.8 |
|  | ftsEX | 317 | 3.5 ± 1.5 |
|  | *ftsE^D162N^X* | 67 | 16.8 ± 6.0 |

^a^ The average length refers to the length of cell chains.
