# Supplementary material for: Roles of ATP Hydrolysis by FtsEX and Interaction with FtsA in Regulation of Septal Peptidoglycan Synthesis and Hydrolysis
Source: mBio. 2020 Jul 7;11(4):e01247-20. doi: 10.1128/mBio.01247-20 (PMC7343993; doi:10.1128/mBio.01247-20)
Supplement: TABLE S6 [file mBio.01247-20-st006.docx]

**Table S6. Length of cells of strain SD523 (*ftsA* ΔftsEX ΔnlpD att^λ^P_BAD_::ftsEX*) and SD524 (*ftsA*^,G366D^ ΔftsEX ΔnlpD att^λ^P_BAD_::ftsEX*) expressing different *ftsEX* alleles after depletion of arabinose.**

| **Arabinose depletion time (h)** | **Genotype** | ***ftsEX* allele** | **# Cells** | **Average length ^a^ ± STDEV (μm)** |
| --- | --- | --- | --- | --- |
| 0 | *ftsA* ΔftsEX ΔnlpD* | - | 270 | 3.8 ±1.2 |
|  |  | ftsEX | 286 | 3.7±1.0 |
|  |  | *ftsE^D162N^X* | 240 | 4.3±1.4 |
|  | *ftsA*^, G366D^ ΔftsEX ΔnlpD* | - | 60 | 14.4±8.3 |
|  |  | ftsEX | 125 | 9.7±4.1 |
|  |  | *ftsE^D162N^X* | 86 | 12±7.0 |
| 3 | *ftsA* ΔftsEX ΔnlpD* | - | 95 | 14.9±5.7 |
|  |  | ftsEX | 248 | 4.2±1.3 |
|  |  | *ftsE^D162N^X* | 47 | 27.0±9.9^b^ |
|  | *ftsA*^, G366D^ ΔftsEX ΔnlpD* | - | 12 | 58.9±37.0 |
|  |  | ftsEX | 271 | 5.2±1.7 |
|  |  | *ftsE^D162N^X* | 175 | 8.3±3.6 |
| 6 | *ftsA* ΔftsEX ΔnlpD* | - | ND^c^ | ND |
|  |  | ftsEX | 323 | 4.3±1.3 |
|  |  | *ftsE^D162N^X* | ND | ND |
|  | *ftsA*^, G366D^ ΔftsEX ΔnlpD* | - | ND | ND |
|  |  | ftsEX | 193 | 6.2±2.2 |
|  |  | *ftsE^D162N^X* | 137 | 10.8±5.5 |

^a^ The average length of cells refers to the length of individual cells or cell chains.

^b^ The average length of cells here were of smooth filamentous cells.

^c^ ND: Not determined. The cells from these samples were too long to be measured.
