# Supplementary material for: Roles of ATP Hydrolysis by FtsEX and Interaction with FtsA in Regulation of Septal Peptidoglycan Synthesis and Hydrolysis
Source: mBio. 2020 Jul 7;11(4):e01247-20. doi: 10.1128/mBio.01247-20 (PMC7343993; doi:10.1128/mBio.01247-20)
Supplement: TABLE S7 [file mBio.01247-20-st007.docx]

**Table S7. Average cell lengths of *ftsA* and ftsA*^, G366D^* strains with or without *nlpD*.**

| **Strain** | **Average cell length ± STDEV (μm) 0.5 % NaCl** | **Average cell length ± STDEV (μm) 1.5 % NaCl** |
| --- | --- | --- |
| *ftsA** | 3.8 **±** 0.9 (51%)^a^ | 3.8 **±** 1.0 (65%) |
| *ftsA*^,G366D^* | 5.2 **±** 1.3 (58%) | 4.8 **±** 1.2 (43%) |
| *ftsA* ΔnlpD* | 3.7 **±** 0.8 (47%) | 3.6 **±** 0.9 (41%) |
| *ftsA*^,G366D^ ΔnlpD* | 10.9 **±** 5.7 (94%) | >25 (100%) |

^a^ The numbers in parentheses are the percentage of dividing cells.
